# Presence and Characterization of Zoonotic Bacterial Pathogens in Wild Boar Hunting Dogs (*Canis lupus familiaris*) in Tuscany (Italy)

**DOI:** 10.3390/ani11041139

**Published:** 2021-04-16

**Authors:** Giovanni Cilia, Filippo Fratini, Barbara Turchi, Valentina Virginia Ebani, Luca Turini, Stefano Bilei, Teresa Bossù, Maria Laura De Marchis, Domenico Cerri, Fabrizio Bertelloni

**Affiliations:** 1 Department of Veterinary Sciences, University of Pisa, Viale delle Piagge 2, 56124 Pisa, Italy; giovanni.cilia@vet.unipi.it (G.C.); filippo.fratini@unipi.it (F.F.); valentina.virginia.ebani@unipi.it (V.V.E.); luca.turini@phd.unipi.it (L.T.); domenico.cerri@unipi.it (D.C.); fabrizio.bertelloni@unipi.it (F.B.); 2 Istituto Zooprofilattico Sperimentale del Lazio e della Toscana M. Aleandri, 00178 Rome, Italy; stefano.bilei@izslt.it (S.B.); teresa.bossu@izslt.it (T.B.); marialaura.demarchis@izslt.it (M.L.D.M.)

**Keywords:** *Leptospira*, *Salmonella*, *Yersinia*, *Listeria*, zoonoses, hunting dogs

## Abstract

**Simple Summary:**

Wildlife is an important reservoir for several zoonotic pathogens, and wild animals can contribute to disease transmission to humans or domestic animals via direct or indirect contact. In the One Health approach, the role of wildlife and the wild environment in the maintenance and spread of zoonoses has great importance. Domestic dogs (*Canis lupus familiaris*) employed in wild boar hunts may be a good indicator to evaluate this. This investigation reports the presence of *Leptospira* spp. and antimicrobial-resistant *Salmonella* spp. and *Yersinia enterocolitica* in wild boar hunting dogs in the Tuscany region (Italy). The results obtained suggest that wildlife may be the source of pathogens detected in dogs; indeed, all pathogens may be carried by wild animals, in particular wild boar. This investigation highlights the possible risk for dogs connected to work activities. Furthermore, considering that humans could be exposed to the same pathogens during outdoor activities, constant monitoring seems necessary to evaluate the transmission risk.

**Abstract:**

Domestic dogs (*Canis lupus familiaris*) used for wild boar (*Sus scrofa*) hunting may represent incidental hosts for several zoonotic pathogens. This investigation aimed to evaluate the presence of anti-*Leptospira* antibodies and the occurrence, antimicrobial resistance, and virulence of *Salmonella* spp., *Yersinia enterocolitica*, and *Listeria monocytogenes* in sera and rectal swabs collected from 42 domestic hunting dogs in the Tuscany region (Italy). Regarding *Leptospira*, 31 out of 42 serum samples (73.8%) were positive and serogroup Pomona was the most detected (71.4%) at titers between 1:100 and 1:400. Four *Salmonella* isolates (9.52%) were obtained, all belonging to serotype Infantis; two of them showed antimicrobial resistance to streptomycin, while *pipB* and *sopE* presence was assessed in all but one isolate. Concerning *Yersinia enterocolitica*, seven isolates (16.7%) were obtained, six belonging to biotype 1 and one to biotype 4. Resistance to amoxicillin–clavulanic acid, cephalothin, and ampicillin was detected. Biotype 4 presented three of the virulence genes searched (*ystA*, *ystB*, *inv*), while isolates of biotype 1 showed only one gene. No *Listeria monocytogenes* was isolated from dog rectal swabs. The results suggest that hunting dogs are exposed to different bacterial zoonotic agents, potentially linked to their work activity, and highlight the possible health risks for humans.

## 1. Introduction

The coexistence between domestic dogs (*Canis lupus familiaris*) and humans has lasted for about 40,000 years. By this relationship, dogs have evolved thanks to several domestication events [1,2]. During these multiple events, dogs did not modify their complex body language, and humans used their sophisticated forms of social cognition and communication to train them [1,2]. The training led to dividing dogs into working-class categories and employing them in jobs that help humans in several areas, including hunting.

Traditionally in Italy, domestic hunting dogs have been employed by hunters to track wildlife, as well as wild boar (*Sus scrofa*), during the activity known as a “drive hunt”, in in which several dogs are used [3].

Due to contact with some wild animals species and the sharing of environmental areas, domestic hunting dogs can be affected by a large variety of pathogens [4,5,6,7,8,9,10,11]. Moreover, wild boar hunting dogs during game activities or at the end of slaughtering are usually fed raw wild boar meat and/or slaughter waste, such as liver, lung, spleen, heart, kidney, and sometimes testicles; this practice can increase the risk of disease transmission [9].

Wildlife acts as a reservoir for pathogens that contribute to maintaining and/or disseminating important infectious diseases [12,13,14,15,16,17]. Regarding Tuscany (Italy), among bacterial zoonoses, different etiological agents have been detected in wild animals, in particular *Leptospira*, *Salmonella*, *Yersinia*, and *Listeria* [18,19,20,21,22,23,24,25,26,27].

Leptospirosis is a neglected and re-emerging zoonosis caused by Gram-negative bacteria belonging to the *Leptospira* genus [28]. *Leptospira* infection, which is widespread worldwide, is maintained by a large variety of domestic and wild animal species, which act as asymptomatic maintenance hosts [29,30]. The localization of renal reservoirs contributes to maintaining the infection in the environment through constant shedding of *Leptospira* in urine. In this way, accidental contact with *Leptospira*-infected urine causes an incidental infection that could lead to clinical disease, as reported in dogs [30,31].

Salmonellosis is the second most diffused zoonosis in Europe [32]. It is caused by a Gram-negative rod-shaped, flagellated, and facultative anaerobic bacteria belonging to the *Salmonella enterica* species, which includes more than 2600 serovars [33]. *Salmonella enterica* strains can cause illnesses in both humans and animals [33,34].

*Yersinia enterocolitica* is the etiological agent of yersiniosis, another important zoonosis [32]. Bacteria belonging to this species can survive in the environment for a long time. *Yersinia enterocolitica* has been divided into more than 70 serotypes, based on differences in the structure of the somatic antigen, and into 6 biotypes based on its biochemical characteristics [35].

Listeriosis is a zoonosis caused by *Listeria monocytogenes*, a Gram-positive and facultative intracellular bacterium [36]. *Listeria* infection occurs as an epidemic or sporadically, and more than 90% of human cases worldwide have generally been caused by strains belonging to serovars 1/2a, 1/2b, and 4b of 13 possible serovars [37].

All of these foodborne zoonoses are spread worldwide and diffused in several environments, such as soil, water, feces, and meat [38]. Moreover, one of the main forms of *Salmonella* spp. and *Yersinia enterocolitica* transmission is the consumption of domestic and wild swine meat [39,40].

This investigation was aimed at evaluating infection by *Leptospira* spp., *Salmonella* spp., *Yersinia enterocolitica*, and *Listeria monocytogenes* in domestic hunting dogs employed for wild boar hunting. *Leptospira* prevalence was analyzed using serological assay, while *Salmonella* spp., *Yersinia enterocolitica*, and *Listeria monocytogenes* were investigated through isolation methods. The presence of virulence genes and antimicrobial resistance of the obtained isolates was evaluated.

## 2. Materials and Methods

### 2.1. Sampling

In January 2020, at the end of the hunting season that started in November 2019, we collected radial vein blood samples and rectal swabs from domestic hunting dogs (*Canis lupus familiaris*) employed in wild boar hunting in the provinces of Pisa and Lucca (Tuscany, Italy). All specimens were sampled from hunting dogs belonging to hunters who collaborated with the authors during sample collection for previous research [18,22,23,41,42].

Samples were collected after authorization by the Organismo Preposto al Benessere degli Animali (OPBA) of the University of Pisa with protocol no. 21/2020. During the sampling phase, which took place where the dogs were housed by their owners, information about vaccination programs and vaccines used for leptospirosis was recorded. In addition, the occurrence of previous gastrointestinal disorders was taken into consideration, and subjects with recent or previous intestinal diseases were not included in the study.

Blood samples were centrifuged at 10,000 rpm for 10 min to obtain serum. The sera were kept at −20 °C until use for the serological test. Rectal swabs were processed right away to obtain isolates.

### 2.2. Microscopic Agglutination Test (MAT)

To provide evidence of the vaccine’s effectiveness and to investigate the possible native response against *Leptospira* serogroups not covered by vaccines, a serological analysis was carried out. In particular, to detect *Leptospira* antibodies, sera were tested by the microscopic agglutination test (MAT) [43]. A titer of 1:100 was considered positive. The following live *Leptospira* antigens were used for the MAT: *Leptospira interrogans* serovar Icterohaemorrhagiae (serogroup Icterohaemorrhagiae, strain RGA), *L. interrogans* serovar Canicola (serogroup Canicola, strain Alarik), *L. interrogans* serovar Pomona (serogroup Pomona, strain Mezzano), *L. kirschneri* serovar Grippotyphosa (serogroup Grippotyphosa, strain Moskva V), *L. borgpetersenii* serovar Tarassovi (serogroup Tarassovi, strain Mitis Johnson), *L. interrogans* serovar Bratislava (serogroup Australis, strain Riccio 2), *L. interrogans* serovar Hardjo (serogroup Sejroe, serovar Hardjoprajitno), and *L. borgpetersenii* serovar Ballum (serogroup Ballum, strain Mus 127).

### 2.3. Bacterial Isolation and Characterization

From rectal swabs, *Salmonella* spp., *Yersinia enterocolitica*, and *Listeria monocytogenes* isolation was performed as previously described [22,44]. *Salmonella* spp. isolates were serotyped by slide agglutination test with commercial antisera (Statens Serum Institut, Copenhagen, Denmark), according to the Kauffmann–White scheme. *Yersinia enterocolitica* isolates were characterized based on biochemical tests to distinguish the biotype [45].

### 2.4. Antimicrobial Resistance

The antimicrobial susceptibility of all obtained isolates was evaluated using the disc diffusion test on Mueller Hinton Agar (Oxoid, Ltd., Basingstoke, UK) [46]. The following antibiotics (Oxoid) were employed: amoxicillin–clavulanic acid (AMC; 30 µg), ampicillin (AMP; 10 µg), aztreonam (ATM; 30 µg), cephalothin (KF; 30 µg), cefotaxime (CTX; 30 µg), cefoxitin (FOX; 30 µg), chloramphenicol (C; 30 µg), enrofloxacin (ENR; 5 µg), gentamycin (CN; 10 µg), imipenem (IPM; 10 µg), nalidixic acid (NA; 2 µg), nitrofurantoin (F; 300 µg), streptomycin (S; 10 µg), sulfamethoxazole–trimethoprim (STX; 25 µg), and tetracycline (TE; 30 µg). CLSI (Clinical and Laboratory Standards Institute) zone diameter interpretive criteria were used [47].

### 2.5. Virulence Genes

DNA was extracted from overnight bacterial cultures of each isolate using Quick-DNA Plus Kits (Zymo Research, Irvine, CA, USA) following the manufacturer’s guidelines.

In *Salmonella* spp. isolates, the presence of *mgtC*, *pipB*, *sopB*, *spvR, spvC*, *gipA*, *sodCI*, and *sopE* genes, linked to virulence, was evaluated using primers and protocols as previously reported [48,49,50,51,52].

In *Yersinia enterocolitica* isolates, the presence of the following virulence genes was evaluated using previously published primers and protocols: *ail*, *virF*, *ystA*, *ystB*, and *inv* [53,54,55].

Each polymerase chain reaction (PCR) was carried out in a total volume of 50 μL, including 25 μL of EconoTaq PLUS 2× Master Mix (Lucigen Corporation, Middleton, WI, USA), 0.5 μM of each primer, 3 μL of DNA, and distilled water to reach the final volume. An automated thermal cycler Gene-Amp PCR System 2700 (PerkinElmer, Norwalk, CT, USA) was employed to perform the amplifications, consisting of initial denaturation at 95 °C for 10 min, 35 cycles of denaturation at 95 °C for 1 min, annealing for 2 min, extension at 72 °C for 1 min, and a final extension at 72 °C for 10 min. Annealing temperatures were set based on the specific primers employed. PCR products were analyzed by electrophoresis on 1.5% agarose gel at 100 V for 45 min. PCR Sizer 100 bp DNA Ladder (Norgen Biotek, Thorold, ON, Canada) was used as a DNA marker.

### 2.6. Statistical Analysis

Data were reported on Excel (Microsoft, Albuquerque, NM, USA) and analyzed with the chi-square (X^2^) test. The statistical test was used to evaluate the infection rate of each pathogen in relation to sex (male or female) and hunting province (Pisa or Lucca). The statistical significance threshold was set at *p* ≤ 0.05 [56], and a 95% confidence interval was calculated.

## 3. Results

Blood and rectal swabs were collected from 42 hunting dogs, 30 from a hunting company in Pisa and 12 from Lucca; in particular, samples were collected from dogs belonging to 10 different owners (Table 1). All dogs were housed in a single box, except during work activities or training. The box appeared clean and in good condition; owners noted that rats and mice were not present, but they did not have a regular rat-control program. All animals were fed only with commercial feed. All dogs (22 males, 20 females) were healthy and did not show clinical signs of leptospirosis or gastrointestinal disorder at sampling time. The vaccines given to the investigated dogs were Eurican L-multi^®^ (including serovars Canicola, Icterohaemorrhagiae, and Grippotyphosa), Nobivac L-4^®^ (including serovars Canicola, Icterohaemorrhagiae, Bratislava, and Grippotyphosa), and Canigen DHPPi/L^®^ (including serovars Canicola and Icterohaemorrhagiae), as reported in Table 1.

### 3.1. Leptospira spp.

Overall, all sera but one were positive by MAT (Table 1). Some of the positive reactions against Icterohaemorrhagiae, Canicola, Australis, and Grippotyphosa serogroups, 31 out of 42 serum samples (73.8%; 95% confidence interval (CI): 60.5–87.1%), probably linked to vaccination, were positive to serological analysis (shown in bold in Table 1).

Pomona was the most-recorded serogroup (71.4%; 95% CI: 57.7–85.0%), serologically detected in most of the positive sera (30/31). Among them, 8 (26.7%; 95% CI: 10.8–42.5%) showed a titer of 1:400, 17 (56.7%; 95% CI: 38.9–74.4%) a titer of 1:200, and 5 (16.7%; 95% CI: 3.3–30.0%) a titer of 1:100. One serum was also positive to serogroup Australis at a titer of 1:100. Finally, two sera (6.7%; 95% CI: 0.0–15.6%) were positive to serogroup Grippotyphosa, one at a titer of 1:100 and one at 1:200.

No statistical differences (*p* > 0.05) were reported for serological positivity considering hunting company, province, and dog sex.

### 3.2. Salmonella spp.

Four *Salmonella* strains (9.52%; 95% CI: 0.6–18.3%) were isolated from hunting dog rectal swabs. All isolates belonged to *Salmonella enterica* subspecies *enterica* serotype Infantis. Only two of them showed resistance to streptomycin (50.0%). All but one isolate harbored some virulence genes. Two isolates scored positive only to the *pipB* gene, and one to *pipB* and *sopE* (Table 2).

No statistical differences (*p* > 0.05) were reported for the number of obtained *Salmonella* spp. isolates considering hunting company, province, and dog sex.

### 3.3. Yersinia enterocolitica

In total, seven strains of *Yersinia enterocolitica* (16.7%; 95% CI: 5.4–27.9%) were isolated, six of which were biochemically confirmed as biotype 1, and one as biotype 4 (Table 3).

All strains were resistant to at least two antimicrobials. In particularly, all strains (100%) were resistant to ampicillin and cephalothin. Moreover, ampicillin resistance was reported in six isolates (85.7%), cefoxitin resistance in two isolates (28.6%), and streptomycin, nalidixic acid, and chloramphenicol resistance in one isolate (14.3%).

All but one isolate presented at least one virulence gene; only one isolate, biotype 4, scored positive for more than one gene. The most detected genes were *ystA*, *ystB*, and ail in two out of the seven isolates (28.6%), followed by *inv* and *virF*, detected in one isolate (14.3%).

No statistical differences (*p* > 0.05) were reported for the number of obtained *Yersinia enterocolitica* isolates considering hunting company, province, and dog sex.

### 3.4. Listeria monocytogenes

No *Listeria monocytogenes* isolates were obtained from hunting dog rectal swabs.

## 4. Discussion

This investigation reports infection by *Leptospira* spp., *Salmonella* ser. Infantis, and *Yersinia enterocolitica* in a sample of hunting dogs employed in wild boar hunting in two provinces of Tuscany. To the best of the authors’ knowledge, no other investigations have been performed to research these zoonotic bacterial pathogens in Italian hunting dogs.

Concerning *Leptospira* serological results, all dogs but one were positive, at least for one serogroup. Some positive reactions could be related to immune response induced by vaccine administration, as previously reported [57,58,59]. Indeed, in all serum samples, titers of 1:100 and 1:200 were reported in vaccinated dogs for serovars included in the vaccine. In detail, antibodies for serogroup Icterohaemorrhagiae, Canicola, Grippotyphosa, and Australis were found in dogs regularly vaccinated with Nobivac L-4^®^ (MSD Animal Health); for serogroup Icterohaemorrhagiae, Canicola, and Grippotyphosa in dogs vaccinated with Eurican L-multi^®^ (Boehringer Ingelheim Animal Health); and for serogroup Icterohaemorrhagiae and Canicola in dogs vaccinated with Canigen DHPPi/L^®^ (Virbac S.r.l.). Serogroup Pomona was found in 71.4% of sampled hunting dogs, at titers from 1:100 to 1:400. The serological titers suggested chronic or very recent infection, probably mediated by vaccine status.

Pomona is a serogroup strictly connected to pigs. Indeed, *Leptospira*, which belongs to this serogroup, has been widely detected in the Tuscany region in domestic and wild swine [19,60,61], particularly in wild boar sampled in the same area and the same period [23]. Pomona was recently isolated in Tuscany from a crested porcupine [21]; even if the role of this animal as maintenance or accidental host is not still clear, this suggests a link to animals other than wild boar. Due to the rare Pomona infection reported in dogs, this serovar is not included in dog vaccines [57], even though infection causes severe disease with lethargy, fever, inappetence, diffuse hemorrhage, and renal and liver failure [62,63]. In recent years in Italy, Pomona incidence in dogs has increased [64,65,66,67], probably due to contact with wild boar, which play a role in spreading the disease as a reservoir.

Two serum samples showed a positive reaction to Grippotyphosa live antigen, at titers of 1:100 and 1:200. In Europe, this is an emerging serogroup in dogs [65,67,68,69,70] and is included in the dog leptospirosis vaccine [57]. In two serum samples, *Leptospira* co-infection was reported: one was positive to Grippotyphosa and Pomona at titers of 1:200 and 1:100, respectively, and the other to Pomona and Australis at titers of 1:200 and 1:100, respectively. It cannot be excluded that the co-infection could be related to an unspecific reaction between the serum antibody and *Leptospira* antigens [28]. Grippotyphosa and Australis infection could be related to direct or indirect contact with small rodents, lagomorphs, and hedgehogs, which act as reservoirs for these serovars and are particularly abundant among the wildlife population in this area [65]. Finally, although serology has high diagnostic value for leptospirosis and MAT is considered the gold standard test, with high sensitivity and specificity, in order to better understand the real risk for hunting dogs, it will be necessary to perform isolation or molecular investigation on urine and blood samples in the future.

Regarding rectal swabs, 9.52% were positive to *Salmonella* spp. Although no studies have been performed on hunting dogs, the prevalence found in this investigation is low, as previously reported in studies on domestic dogs [5,71,72]. All of them belonged to *Salmonella enterica* subspecies *enterica* serotype Infantis. This serotype is usually associated with swine [73,74] and has been isolated in free-ranging wild boar in the same investigated area [22] and in the north of Italy [75]. However, other sources of infection exist other than wild swine; indeed, *S.* ser Infantis was also detected in wild carnivores, ruminants, and birds in Italy [76]. Although no statistical differences emerged regarding *Salmonella* positivity, all infected dogs were from the same owner. For this reason, we cannot exclude that infection was not linked to work activities, but to the environment where the animals live or the management of these dogs. Only two *Salmonella* isolates were resistant to antimicrobials, and particularly only to streptomycin. Streptomycin resistance is well documented in *Salmonella*, particularly in strains isolated from dogs [77,78] and swine [76,79,80]. Concerning virulence genes, *pipB* and *sopE* were the only two detected, found only in wild boar sampled in Tuscany [22]. Both genes were found in the S395 isolates, while only *pipB* was found in in S397 and S398. The *sopE* gene is transmitted by phage and is involved in the invasion of intestinal epithelial cells. It also stimulates the inflammatory response in the host [44]. The *pipB* gene is associated with *Salmonella* pathogenicity island 5 (SPI-5) and plays a role in the survival of *Salmonella* in the intracellular environment [81].

During this investigation, *Yersinia enterocolitica* was isolated from 16.7% of rectal swabs from hunting dogs. As for *Salmonella*, no studies have been carried out on *Yersinia enterocolitica* infection in hunting dogs. A very close prevalence was previously detected in domestic dogs [82,83,84] and in wild boar hunted in the same Italian region [22]. The most diffuse is biotype 1, while only one isolate belongs to biotype 4. These data are in accordance with the prevalence of yersiniosis and biotypes distribution in wild boar [22,85,86]. As for *Salmonella*, other sources of infection cannot be definitively excluded; although *Y. enterocolitica* has been isolated from resident and migratory wild fauna in Italy [87,88], no recent data on the investigated area are available to allow a robust hypothesis. Among isolates belonging to biotype 1, only one virulence gene (within *ail*, *ystA*, *ystB*, and *virF*) was detected, while in biotype 4 the *ystA, ystB*, and *inv* genes were detected. As previously reported in swine [85,89] and wild boar [22], high resistance to amoxicillin–clavulanic acid and cephalothin was also detected in isolates from investigated dogs, but this result could be linked to intrinsic resistance, as previously reported [90]. The rates of resistance to nalidixic acid, chloramphenicol, and streptomycin found in *Yersinia enterocolitica* isolates from dogs seem to be similar to the antimicrobial resistance associated with wild boar strains [22].

The high prevalence of *Leptospira interrogans* serogroup Pomona detected serologically seems to be connected to the infection circulating in free-ranging wild boar in the Tuscany region [19,23,65]. The same association could be hypothesized for *Salmonella* spp. and *Yersinia enterocolitica*, comparing the results of this investigation with the infection of wild boar sampled in the same investigated area, although other sources of infection cannot be excluded. All dogs were healthy at the time of sampling, and the owners did not report previous disease linked to the investigated pathogens; hunting dogs could potentially become asymptomatic reservoirs and shedders of these bacteria, contributing to their diffusion and representing a possible danger for the owners. This investigation highlights some of the risks hunting dogs are exposed to, presumably linked to their work activities, and the potential hazard for humans sharing the same environment for work or recreational activities.

## 5. Conclusions

In conclusion, the results of this investigation suggest that hunting dogs could be exposed to different pathogens, potentially during their work activities. Indeed, some of these pathogens are often associated with wildlife, in particular wild boar. To provide strong evidence on the sources of these pathogens, the number of hunting companies and dogs should be increased and a deep investigation into wildlife and the wild environment should be carried out to obtain an important number of isolates to be compared with phenotypic and molecular methods.

## Figures and Tables

**Table 1:** Serological reactions detected in hunting dog sera in relation to *Leptospira* serogroups, their vaccination state, and hunting company.

Dog	Sex	Year	Breed	Hunting Company	*Leptospira* Serogroup								Vaccine
					Ic	Ca	Po	Gr	Ta	Au	Se	Ba	
D1 *	M	2	SM	Pisa	1:100	1:100	**1:400**	1:100					E
D2 *	F	6	SM	Pisa	1:100	1:100		1:100					E
D3 *	F	2	SM	Pisa	1:100	1:100	**1:200**	1:100					E
D4 *	M	2	SM	Pisa	1:100	1:100		1:200					E
D5 *	M	2	SM	Pisa	1:100	1:100	**1:100**	1:100					E
D6 *	F	9	ES	Pisa	1:100	1:100	**1:100**	1:100					E
D7 *	M	6	ES	Pisa	1:100	1:100	**1:100**	1:100					E
D8 *	F	6	SM	Pisa	1:100	1:100	**1:200**	1:100					E
D9 *	F	4	SM	Pisa	1:100	1:100	**1:400**	1:200					E
D10 *	M	4	SM	Pisa	1:200	1:100	**1:400**	1:100					E
D11 *	F	10	SM	Pisa	1:100	1:100		1:100					E
D12 *	F	2	SM	Pisa	1:100	1:100		1:200					E
D13 *	F	1	SM	Pisa	1:100	1:100	**1:400**	1:100					E
D14 *	F	1	SM	Pisa	1:100	1:100	**1:200**	1:100					E
D15 *	F	2	SM	Pisa	1:100	1:100		1:100					E
D16 *	F	3	SM	Pisa	1:100	1:200	**1:400**	1:100					E
D17 ^+^	M	2	ESS	Pisa	1:100	1:100	**1:200**	1:200					E
D18 ^+^	F	7	ESS	Pisa	1:200	1:100	**1:200**	1:100					E
D19 ^¤^	M	4	SM	Pisa	1:100	1:200	**1:200**	1:100		1:100			N
D20 ^¤^	F	3	SM	Pisa			**1:200**						
D21 ^¤^	M	2	SM	Pisa			**1:200**						
D22 ^°^	M	9	BS	Pisa	1:100	1:100	**1:400**	1:100		1:100			N
D23 ^°^	F	3	ESS	Pisa	1:100	1:100	**1:200**	1:100					E
D24 ^#^	M	6	BS	Pisa	1:100	1:100							C
D25 ^#^	M	3	SM	Pisa			**1:200**						
D26 ^#^	M	3	SM	Pisa			**1:200**						
D27 ^#^	M	3	SM	Pisa			**1:400**						
D28 ^#^	M	2	SM	Pisa			**1:200**						
D29 ^#^	F	3	SM	Pisa			**1:200**						
D30 ^#^	F	8	SM	Pisa			**1:200**						
D31	M	1	SM	Lucca	1:100	1:100	**1:200**	1:200		1:100			N
D32 ^•^	M	2	SM	Lucca	1:100	1:100	**1:400**	1:100		1:100			N
D33 ^•^	M	11	GF	Lucca	1:100	1:100		1:100		1:100			N
D34 ^•^	F	2	GF	Lucca	1:100	1:100		1:100		1:100			N
D35 ^•^	M	3	SM	Lucca	1:100	1:100	**1:100**	1:200		1:100			N
D36 ^•^	M	3	SM	Lucca	1:100	1:100		1:100		1:100			N
D37 ^•^	F	7	SM	Lucca	1:100	1:100		1:100		1:100			N
D38 ^◊^	M	7	HB	Lucca			**1:200**						
D39 ^◊^	F	4	SM	Lucca			**1:200**			**1:100**			
D40 ^◊^	F	4	SM	Lucca			**1:100**	**1:200**					
D41 ^□^	M	6	SM	Lucca									
D42 ^□^	M	5	SM	Lucca				**1:200**					

Dog IDs with same symbols from same owners. SM, Segugio Maremmano; ES, English Setter; ESS, English Springer Spaniel; BS, Brittany Spaniel; GF, Griffon Bleu de Gascogne; HB, half-breed; Ic, Icterohaemorrhagiae; Ca, Canicola; Po, Pomona; Gr, Grippotyphosa; Ta, Tarassovi; Au, Australis; Se, Sejroe; Ba, Ballum; E, Eurican L-multi^®^, covering for serovars Canicola, Icterohaemorrhagiae, and Grippotyphosa; N, Nobivac L-4^®^, covering for serovars Canicola, Icterohaemorrhagiae, Bratislava, and Grippotyphosa; C, Canigen DHPPi/L^®^, covering for serovars Canicola and Icterohaemorrhagiae.

**Table 2:** Virulence genes and antimicrobial resistance profiles of analyzed and characterized *Salmonella* spp. strains.

Isolate	Serotype	Dog	Virulence Gene Profile	Antimicrobial Resistance Profile
S395	Infantis	D2	*pipB, sopE*	Streptomycin
S396	Infantis	D9	*-*	-
S397	Infantis	D13	*pipB*	-
S398	Infantis	D16	*pipB*	Streptomycin

**Table 3:** Virulence genes and antimicrobial resistance profiles of analyzed *Yersinia enterocolitica* isolates.

Isolate	Biotype	Dog	Virulence Gene Profile	Antimicrobial Resistance Profile
YD1	1	D6	*ail*	AMP, KF
YD2	1	D7	*ystA*	AMP, AMC, KF, FOX
YD3	1	D10		AMP, AMC, KF
YD4	4	D13	*ystA, ystB, inv*	AMP, AMC, KF, FOX, C, S, NA
YD5	1	D15	*ail*	AMP, AMC, KF
YD6	1	D16	*ystB*	AMP, AMC, KF
YD7	1	D17	*virF*	AMP, AMC, KF

AMC, amoxicillin–clavulanic acid; AMP, ampicillin; KF, cephalothin; FOX, cefoxitin; S, streptomycin; NA, nalidixic acid; C, chloramphenicol.

## Data Availability

The data presented in this study are available within the article.
